# Parental Mental Health and Hostility Are Associated With Longitudinal Increases in Child Internalizing and Externalizing Problems During COVID-19

**DOI:** 10.3389/fpsyg.2021.706168

**Published:** 2021-07-29

**Authors:** Jennifer E. Khoury, Hargun Kaur, Andrea Gonzalez

**Affiliations:** ^1^Department of Psychology, Mount Saint Vincent University, Halifax, NS, Canada; ^2^Department of Psychiatry and Behavioural Neurosciences, McMaster University, Hamilton, ON, Canada; ^3^Offord Centre for Child Studies, McMaster University, Hamilton, ON, Canada

**Keywords:** COVID-19, internalizing, externalizing, maternal mental health, parenting

## Abstract

Children are at high risk for negative COVID-19 related outcomes. The present longitudinal study assessed (1) changes in child internalizing and externalizing problems from before to during the pandemic and (2) whether parent mental health (depression, anxiety, stress) or parenting behavior during COVID-19 were associated with changes in child mental health problems. Sixty eight mother-child dyads participated in this study. Children were approximately five years-old at the time of enrollment and were between the ages of 7–9 years old at the time of the follow-up survey. Parenting behavior, parental depression, anxiety, perceived stress and child internalizing and externalizing problems were measured using validated questionnaires. Children experienced greater internalizing (*t* = 6.46, *p* < 0.001) and externalizing (*t* = 6.13, *p* < 0.001) problems during the pandemic compared to before the pandemic. After taking into account child gender and COVID-related stressors, parental hostility was uniquely associated with greater changes in externalizing problems (β = 0.355, *SE* = 0.178, *p* < 0.05), while maternal anxiety was associated with greater increases in internalizing problems (β = 0.513, *SE* = 0.208, *p* < 0.05). Findings highlight the need for mental health supports for families to limit the impact of the COVID-19 pandemic on child and parent mental health.

## Introduction

The COVID-19 pandemic and the associated public health directives (e.g., school closures, stay-at-home orders) have caused significant disruptions to daily life. Individuals are experiencing elevated rates of psychological distress and mental health challenges (Pierce et al., 2020; Daly et al., 2021). The United Nations named children as one of the most vulnerable groups at greatest risk for COVID-19 related negative outcomes (United Nations, 2020). Despite this, there is a considerable unequal ratio of research examining the impact of the pandemic on adult mental health as compared to child mental health (Racine et al., 2020). There is a dire need to understand how children's mental health is affected by the pandemic and to identify specific factors associated with child mental health problems, in order to determine how to best support children in adjusting to the disruptions brought by the pandemic. To address these needs, the current longitudinal study examines (1) whether child internalizing and externalizing problems have changed from before to during the COVID-19 pandemic and (2) how parental mental health, distress and parenting behavior are associated with any changes in child mental health.

Research indicates that children and adolescents are experiencing high rates of mental health difficulties during the pandemic (Bignardi et al., 2020; Bikmazer et al., 2020; Crescentini et al., 2020; Cusinato et al., 2020; Francisco et al., 2020; Xie et al., 2020), including internalizing problems, such as depression and anxiety (Bignardi et al., 2020), and externalizing problems, such as anger, irritability, and argumentative behavior (Orgilés et al., 2020). Much of this research was conducted during the initial phase of the pandemic, which often included nation-wide lockdowns (e.g., China, Italy). Therefore, there is a need to examine the enduring effects of this pandemic on child mental health in countries (e.g., Canada, USA) where the effects of COVID-19 were delayed but are ongoing.

Longitudinal research is essential to determine whether children are, in fact, experiencing *elevated* mental health problems during the pandemic (Wade et al., 2020). To our knowledge, only a handful of longitudinal studies have been conducted to date. Children (age 7–11), followed before and during the initial lockdown in the UK, experienced elevated levels of depressive symptoms but no significant difference in anxiety (Bignardi et al., 2020). Compared to before the COVID-19 pandemic, during the lockdown in Italy, a sample of preschool children experienced significantly more anxious/depressed behavior, somatic complaints, and aggressive behavior (Cantiani et al., 2021). Furthermore, in a sample of 10- to 13-year-olds in the Netherlands, children's perceived stress during the lockdown mediated associations between externalizing behavior prior to the lockdown and elevated externalizing behavior during the lockdown (Achterberg et al., 2021). In contrast, a study of predominantly Hispanic/Latinx youth (age 10–14) in the United States (Penner et al., 2020) found reductions in internalizing and externalizing problems from before the spread of COVID-19 in the US (January 2020) to during the pandemic (April-May 2020). In addition, an ecological momentary assessment study did not find significant differences in adolescent negative affect from before (2018–2019) and during the pandemic (Janssen et al., 2020). Considering the conflicting results of previous longitudinal studies, additional research is needed to examine the potential changes in child mental health before and during the pandemic, as well as the relevant risk factors predicting these changes.

Given the elevated rates of parent mental health difficulties and distress, it is also important to consider how parent mental health impacts child mental health during the pandemic. Extensive research documents the close connection between parent and child mental health (e.g., Goodman et al., 2011; Johnston et al., 2013). Different forms of parental mental health (e.g., anxiety, depression) and stress are associated with both child internalizing and externalizing problems (e.g., Goodman et al., 2011; Stone et al., 2016). Indeed, mothers are experiencing high levels of stress, anxiety, and depression during the COVID-19 pandemic (Cameron et al., 2020; Patrick et al., 2020; Adams et al., 2021). Furthermore, during the pandemic, parental depression, anxiety and stress have been associated with higher child and adolescent internalizing symptoms (Crescentini et al., 2020; Whittle et al., 2020) and parental mental health problems and stress were related to psychosocial concerns in preschool children (Davidson et al., 2020). Importantly, research has yet to examine whether parent mental health problems and stress during the pandemic are associated with *changes* in child mental health from before to during the pandemic.

Given that parents are currently experiencing unprecedented levels of stress and mental health difficulties (e.g., Adams et al., 2021), both of which can impact parenting practices (e.g., Rueger et al., 2011), parenting behavior is an additional likely contributor to child mental health during the pandemic. Parenting behavior has been repeatedly associated with child mental health (McLeod et al., 2007; Achtergarde et al., 2015; Ryan et al., 2017). Much of this research, shows that negative parenting behaviors (e.g., hostility, criticism) are linked to more child mental health difficulties, whereas positive parenting behaviors (e.g., support, warmth) are associated with less child mental health problems (for review see Yap et al., 2014). During the COVID-19 pandemic, parental verbal hostility was linked to higher child hyperactivity and inattention (Marchetti et al., 2020). Moreover, parent hostility and warmth were associated with different forms of child internalizing and externalizing behaviors during the pandemic (Whittle et al., 2020). However, it remains unclear whether parental mental health and hostile or supportive parenting practices are related to *changes* in child mental health difficulties from before to during the pandemic.

In addition to parental mental health and parenting behaviors, it is imperative to account for the impact of COVID-specific stressors that families may be experiencing. COVID-specific stressors, including isolation, quarantine, and financial difficulties, have been linked to more parental and child mental health difficulties (Whittle et al., 2020; Khoury et al., 2021) as well as higher levels abusive and neglectful parenting during the pandemic (Calvano et al., 2021; Connell and Strambler, 2021; Lee et al., 2021). Gender is an additional factor that should be considered when examining child mental health outcomes, given research demonstrating gender differences in child internalizing and externalizing problems (Negriff and Susman, 2011; Tompkins et al., 2011; Ara, 2016). Therefore, the current study will account for COVID-related stressors and child gender when examining how parent mental health and parenting behavior impact changes in child internalizing and externalizing problems during the pandemic.

### Aims of Current Study

The present study builds upon prior research by: (1) following a longitudinal cohort of school-aged children from before the pandemic to during the pandemic to examine *changes* in internalizing *and* externalizing problems; (2) assessing how parent mental health (i.e., depression, anxiety, and stress), as well as parenting behavior (i.e., hostility and support) during the pandemic are associated with changes in child mental health; and (3) examining these associations after the acute phase of the pandemic, given that prior research primarily focuses on initial lockdown periods early in the pandemic. We hypothesized that (1) children would experience more internalizing and externalizing problems during the pandemic, compared to before the pandemic; and (2) greater parental mental health problems (depression, anxiety and stress symptoms) as well as less adaptive parenting behaviors (i.e., more hostile parenting and less supportive parenting) will be associated with more severe internalizing and externalizing problems.

## Methods

### Sample

Mother-child dyads from Ontario, Canada, who had previously participated in a longitudinal study (Prime et al., 2020) were contacted to participate in the present COVID-19 follow-up study. Of the 169 contacted families, 54 were unreachable, 17 declined participation, 25 expressed interest in participating but did not begin the survey, five completed only part of the follow-up survey (though were missing multiple questionaries of interest), and 68 completed the COVID-19 follow-up study. Therefore, this is a convenience sample of a larger cohort. At the time of the COVID-19 follow-up, children were between 7 and 9 years old (*M* = 7.87, *SD* = 0.75 years); children were on average 5.15 years old (*SD* = 2.06 years) when they previously participated in this study. See Table 1 for sample characteristics. The COVID-19 cohort did not differ from the larger longitudinal cohort with respect to child sex, race, ethnicity, family income, maternal education, and marital status (*p*_range_ = 0.22–0.93). In addition, the COVID-19 cohort did not differ on baseline levels of internalizing problems (*t* = 1.10, *p* = 0.273), externalizing problems (*t* = 0.575, *p* = 0.566), or maternal depressive symptoms (*t* = 1.49, *p* = 0.137) (maternal anxiety and perceived stress were not collected at baseline).

**Table 1:** Sample characteristics.

	**Sample Characteristics % (N)**
**Child Gender**	
Male	51.5 (35)
Female	47.1 (32)
Transgender	1.5 (1)
**Child Race**	
White	83.8 (57)
Asian	1.5 (1)
Mixed Race	8.8 (6)
Other	5.9 (4)
**Marital status**	
Married	72.1 (49)
Common-law	17.6 (12)
Separated	4.4 (3)
Divorced	2.9 (2)
Widowed	1.5 (1)
Single	1.5 (1)
**Maternal Education**	
Secondary school	8.8 (6)
Community college	25.0 (17)
University (Undergraduate)	29.4 (20)
Postgraduate	36.8 (25)
**Annual Household Income**	
$20,000 to $34,999	4.4 (3)
$35,000 to $69,999	13.2 (9)
$70,000 to $89,999	10.3 (7)
$90,000 to $109,999	13.2 (9)
$110,000 to $149,999	22.1 (15)
$150,000 to $199,999	19.1 (13)
≥ 200,000	17.6 (12)

### Procedure

In the current COVID-19 follow-up study, mothers completed online questionnaires between May and November 2020.^1^ Data was also collected prior to the COVID-19 pandemic (between 2016 and 2018) as part of a larger study, where mothers completed a series of questionnaires about their child's behavior during a home visit. The larger study was approved by the McMaster Research Ethics Board and the St. Joseph's Healthcare Hamilton Research Ethics Board. The follow-up COVID study was approved by the Hamilton Integrated Research Ethics Board under Project #10816.

### Measures

#### Demographic and COVID-19 Experiences

Mothers provided demographic information for themselves and their child. They also provided information about how the COVID-19 pandemic has impacted their household, including financial strain, isolation, health-risk (Khoury et al., 2021).

#### Parent Behavior Inventory (PBI)

During the COVID-19 follow-up survey, mothers completed the 20-item PBI (Lovejoy et al., 1999) to capture parenting behaviors over the past month. The PBI includes a Hostile/Coercive subscale (e.g., threatening child, losing temper with child) and a Supportive/Engaged subscale (e.g., listening to child's feelings, offering to help the child). The Hostile/Coercive subscale (Cronbach's α = 0.82) and the Supportive/Engaged subscale (Cronbach's α = 0.85) showed good internal consistency in the current sample.

#### Centre for Epidemiologic Studies Depression Scale (CES-D)

Mothers completed the 10-item CES-D (Andresen et al., 1994) to assess depressive symptoms over the past week. The CES-D total score ranges from 0 to 30, with a score of 10 or higher indicating the presence of clinically significant depressive symptoms (Andresen et al., 1994).

#### Generalized Anxiety Disorder-7 (GAD-7)

Mothers also completed the seven-item GAD screener which assesses GAD symptoms over the past two weeks (Spitzer et al., 2006). The GAD-7 total score ranges from 0 to 21, a score of 10 or higher indicates possible clinical levels of anxiety.

#### Perceived Stress Scale (PSS)

The 10-item PSS was used to assess mother's experiences of stress over the past month (Cohen and Williamson, 1988). The PSS total score ranges from 0 to 40; scores between 0 and 13 indicate low stress, scores between 14 and 26 indicate moderate stress, and scores between 27 and 40 indicate high perceived stress (Cohen and Williamson, 1988).

#### Child Mental Health

During the COVID-19 follow-up, child behavior problems were measured using the Brief Problem Monitor-Parent form for ages 6–18 years (BPM/6-18; Achenbach et al., 2011). The 19-item BPM is an abbreviated version of the Child Behavior Checklist (CBCL; Achenbach and Ruffle, 2000), it includes 19 identical items from the 100 item CBCL. The BPM has subscales for child internalizing behavior (e.g., worry, unhappiness, fear), externalizing behavior (e.g., aggression, oppositionality, temper), and attention problems (e.g., impulsivity, inattention). The BPM has strong reliability and validity (Piper et al., 2014; Penelo et al., 2017). The current study focused on the BPM internalizing and externalizing scales, which demonstrated good internal consistency (Cronbach's α = 0.73 and 0.79, respectively).

In addition, mothers completed the 100-item CBCL (Achenbach and Ruffle, 2000) for pre-school children aged 1.5 to 5 years during the previous longitudinal study. In this sample, the CBCL demonstrated good internal consistency for internalizing and externalizing subscales (Cronbach's α = 0.86 and 0.92, respectively).

### Data Analysis

#### Data Preparation

Standardized T-scores from the BPM and CBCL internalizing and externalizing were used in the present analyses. To prevent the over-interpretation of differences among scores that are in the low normal range, the BPM T-score floor is 50 (Achenbach et al., 2011). Thus, for accurate comparisons between the BPM and CBCL, CBCL T-scores were also truncated at 50 (Achenbach et al., 2011). Child behavior difference scores were computed by subtracting CBCL T scores (assessed before COVID-19) from BPM T-scores (assessed during COVID-19), with positive scores indicating greater behavior problems during COVID-19.

#### Analyses

Preliminary analyses (i.e., descriptive statistics, bivariate correlations, *T*-tests) were conducted using SPSS 26. Paired sample *T*-tests were used to compare child behavior problems before and during the COVID-19 pandemic. Correlations were conducted to assess bivariate associations between potential covariates (sociodemographic, COVID-19 stressors, timing of survey) and outcomes. Variables with statistical significance or theoretical relevance were included in subsequent regression analyses. Two linear regression analyses were conducted to simultaneously assess the associations between parenting behavior and parental depression, anxiety and stress (all continuous scores) and changes in child internalizing and externalizing problems after controlling for baseline levels of child behavior problems. Linear regression analyses were conducted in Mplus Version 8 using full information maximum likelihood (FIML) and bootstrapping to account for missing data (Fox, 2015). Bias-corrected confidence intervals (CIs) were used; 95% CIs that do not contain zero are significant at *p* < 0.05.

#### Missing Data

7.4% (*k* = 5) of the CBCL from the prior longitudinal study were missing. There was no missing data for the PBI, CES-D-10, or BPM from the current COVID-19 follow up. Based on Little's Missing Completely at Random (MCAR) test, χ^2^(22) = 22.08, *p* = 0.46, this data was deemed to be missing at random and thus appropriate for the use of FIML.

## Results

### Descriptive Statistics

#### COVID-19 Context

On average, mothers completed the survey 103.37 days (*SD* = 25.65 days) after the Ontario provincial government declared a state of emergency in response to the COVID-19 pandemic on March 17, 2020. Between March and June 2020, schools were closed, in July and August a phased re-opening plan occurred, and September to November families had an option to return to in-person school. At the time of the survey, 26.5% (*n* = 18) of mothers reported being under self-quarantine, 26.5% (*n* = 18) reported experiencing a loss of income due to the pandemic, and 72.1% (*n* = 49) reported that their child was moderately to severely upset by COVID-19 restrictions.

#### Maternal Mental Health and Distress

36.8% (*n* = 25) of mothers scored ≥ 10 on the CES-D-10 (*M* = 8.77, *SD* = 6.04), indicating clinically significant levels of depression during the pandemic. Similarly, 26.5% (*n* = 18) of mothers scored ≥ 10 on the GAD-7 (*M* = 6.53, *SD* = 5.47), indicating elevated levels of anxiety. On the PSS (*M* = 17.74, *SD* = 6.62), 26.5% of mothers endorsed low levels of stress, 63.2% moderate stress, and 10.3% high stress.

### Changes in Child Mental Health

Paired sample *T*-tests were used to compare child behavior problems before the pandemic to during the pandemic. Children experienced greater mental health problems during the COVID-19 pandemic compared to before: internalizing T-scores (pandemic: *M* = 57.56, *S*D = 6.87; pre-pandemic: *M* = 52.46, *SD* = 4.84; *t*_(62)_ = 6.46, *p* < 0.001) and externalizing T-scores (pandemic: *M* = 55.30, *SD* = 6.32; pre- pandemic: *M* = 50.95, *SD* = 2.31; *t*_(62)_ = 6.13, *p* < 0.001). This indicates a mean difference of 5.10 in internalizing T-scores and 4.35 in externalizing T scores. *T*-tests were also significant when comparing z scores of internalizing and externalizing problems between baseline and pandemic follow-up.^2^

### Regression Results: Parenting and Maternal Mental Health in Association With Changes in Child Behavior

See Table 2 for Pearson correlations. None of the demographic variables (i.e., child age, gender, race, income, maternal education) were significantly associated with child mental health outcomes. Given the literature indicating gender differences in internalizing and externalizing behavior, gender was retained as a covariate in subsequent regression analyses. In addition, self-quarantine, financial difficulties due to COVID-19, and the number of days since the state of emergency was enacted were co-varied in regression analyses to adjust for the impact of COVID-specific stressors and circumstances on child behavior. Lastly, all regression models controlled for baseline levels of child mental health problems.

**Table 2:** Bivariate correlations among primary study variables and potential covariates.

	**1**	**2**	**3**	**4**	**5**	**6**	**7**	**8**	**9**	**10**	**11**	**12**	**13**	**14**	**15**	**16**	**17**	**18**	**19**	**20**
1. Internalizing change	–																			
2. Externalizing change	0.251^*^	–																		
3. CBCL Internalizing	−0.255^*^	−0.181	–																	
4. CBCL Externalizing	0.023	0.114	0.421^**^	–																
5. BPM Internalizing	0.732^**^	0.101	0.472^**^	0.317^*^	–															
6. BPM Externalizing	0.232^∧^	0.932^**^	−0.008	0.467^**^	0.186	–														
7. CESD-10	0.243^∧^	0.178	0.132	0.011	0.308^*^	0.168	–													
8. PSS	0.253^*^	0.249^*^	0.179	0.094	0.353^**^	0.278^*^	0.643^**^	–												
9. GAD-7	0.373^**^	0.154	0.265^*^	0.069	0.533^**^	0.165	0.689^**^	0.726^**^	–											
10. Parent Support	−0.031	−0.304^*^	−0.113	−0.091	−0.091	−0.244^*^	−0.117	−0.030	−0.090	–										
11. Parent Hostility	0.190	0.492^**^	0.207	0.303^*^	0.348^**^	0.544^**^	0.329^**^	0.314^**^	0.334^**^	−0.272^*^	–									
12. Quarantine	−0.207	−0.195	0.312^*^	0.125	0.112	−0.080	−0.015	0.222^∧^	0.144	0.023	−0.089	–								
13. Financial Difficulties	0.220^∧^	−0.097	0.114	0.155	0.260^*^	−0.025	0.224^∧^	0.150	0.133	−0.007	−0.142	0.048	–							
14. Child Age	−0.050	0.085	0.009	0.175	−0.023	0.132	−0.221^∧^	−0.130	−0.204^∧^	0.052	−0.052	0.017	−0.103	–						
15. Child Gender	−0.106	−0.062	0.041	0.060	−0.014	−0.094	−0.174	−0.177	−0.146	0.071	0.017	0.102	−0.005	0.128	–					
16. Child Race	0.082	0.087	−0.058	−0.047	0.052	0.029	−0.108	−0.058	−0.088	−0.136	−0.073	−0.066	0.093	−0.047	−0.051	–				
17. Household Income	−0.103	0.106	0.120	−0.004	−0.016	0.071	−0.079	0.116	0.020	0.133	0.133	0.161	−0.307^*^	−0.290^*^	0.243^*^	0.032	–			
18. Marital Status	0.043	−0.154	−0.109	−0.058	−0.037	−0.155	0.110	−0.072	−0.072	0.048	−0.279^*^	−0.168	0.348^**^	0.033	−0.168	0.220^∧^	−0.321^**^	–		
19. Parent Education	−0.176	−0.071	−0.062	−0.056	−0.199	−0.098	−0.244^*^	−0.037	−0.157	0.141	0.134	−0.066	−0.532^**^	−0.151	0.116	−0.091	0.397^**^	−0.183	–	
20.Days Between Survey	−0.057	0.112	0.023	0.153	−0.058	0.158	−0.233^∧^	−0.202	−0.252^*^	0.039	−0.064	−0.003	−0.124	0.904^**^	0.059	−0.123	−0.240^∧^	0.053	−0.176	–
21. Days Since State of Emergency	0.117	−0.048	0.185	0.012	0.215^∧^	−0.010	0.173	0.165	0.230^∧^	−0.059	0.179	0.032	−0.101	0.157	−0.125	0.100	−0.112	0.070	−0.026	0.293^*^

*CES–D-10, 10 item Centre for Epidemiologic Studies Depression Scale, completed during the pandemic; GAD-7, 7 item Generalized Anxiety Disorder, completed during the pandemic; PSS, 10 item Perceived Stress Scale, completed during the pandemic; CBCL, Child Behavior Checklist, completed prior to the pandemic; CBCL Internalizing, CBCL Internalizing scale T score; CBCL Externalizing, CBCL Externalizing scale T score; BP, Brief Parent Monitor form, completed during the pandemic; BPM Internalizing, BPM Internalizing scale T score; BPM Externalizing, BPM Externalizing scale T score; Quarantine = 1= under quarantine, 0= not under quarantine; Financial difficulties = financial difficulties during COVID-19 pandemic*.

** *p < 0.01*,

* *p < 0.05*,

∧ *p < 0.10*.

Two linear regression analyses were conducted to predict changes in internalizing and externalizing problems (Table 3). The model predicting changes in child externalizing problems was significant, *F*_(10, 52)_ = 2.83, *p* < 0.01, adjusted *R*^2^ = 0.352. Only parental hostility was significantly associated with greater changes in externalizing behavior from before to during the pandemic; parental support and parent mental health or distress were not significant (Table 3). The model predicting changes in child internalizing problems was also significant, *F*_(10, 52)_ = 3.15, *p* < 0.01, adjusted *R*^2^ = 0.377. More financial problems during the pandemic and higher levels of maternal anxiety during the pandemic was associated with greater increase in child internalizing problems. Other parental mental health measures and parenting behavior were not significant (Table 3).

**Table 3:** Linear regression results: parental mental health, stress, and parenting behavior in association with changes in child behavior problems.

	**Child Internalizing**			**Child Externalizing**		
	**β**	**Std. Error**	**Bootstrap 95% CI**	**β**	**Std. Error**	**Bootstrap 95% CI**
Child gender	−0.022	0.117	−0.252, 0.220	−0.016	0.132	−0.290, 0.217
Days since state of emergency	0.102	0.101	−0.109, 0.295	−0.141	0.132	−0.362, 0.154
Self-quarantine	−0.105	0.109	−0.331, 0.112	−0.205	0.134	−0.447, 0.060
COVID−19 financial difficulties	0.272^*^	0.132	0.014, 0.531	−0.087	0.143	−0.355, 0.206
Child pre-pandemic internalizing/externalizing	−0.415^**^	0.117	−0.638, −0.176	0.005	0.182	−0.336, 0.386
Maternal depression (CES-D)	−0.176	0.187	−0.542, 0.186	−0.079	0.183	−0.408, 0.301
Maternal anxiety (GAD)	0.513^*^	0.208	0.069, 0.900	−0.109	0.180	−0.467, 0.241
Maternal perceived stress (PSS)	−0.042	0.217	−0.434, 0.423	0.346	0.223	−0.166, 0.716
Parent Hostility	0.204	0.136	−0.063, 0.486	0.355^*^	0.178	0.006, 0.719
Parent Support	0.028	0.121	−0.221, 0.251	−0.214^∧^	0.122	−0.482, 0.004

*CES-D, centre for epidemiologic studies depression scale, completed during the pandemic; GAD, generalized anxiety disorder, completed during the pandemic; PSS, perceived stress scale. Child gender dichotomized for regression analyses*.

* *p < 0.05*,

** *p < 0.01*,

∧ *p < 0.10*.

## Discussion

To our knowledge, this is the first longitudinal study assessing the combined impact of maternal mental health and parenting behavior in association with changes in school-aged children's internalizing and externalizing behaviors during the COVID-19 pandemic. Findings indicate significant increases in child internalizing and externalizing problems from before (2016–2018) to during the pandemic (2020). Furthermore, after controlling for baseline mental health problems, maternal hostility during the pandemic was associated with a greater increase in child externalizing behaviors, and maternal anxiety was associated with a greater increase in child internalizing behaviors. These findings reveal that children are experiencing more internalizing and externalizing problems during the COVID-19 pandemic and that parental mental health and parenting practices are important correlates of these observed elevations.

These findings extend cross-sectional research conducted in the early phases of the pandemic (Crescentini et al., 2020; Cusinato et al., 2020; Xie et al., 2020) by demonstrating that the severity of child internalizing and externalizing problems are, in fact, higher than before the pandemic. These findings corroborate a handful of prior longitudinal studies (Bignardi et al., 2020; Achterberg et al., 2021; Cantiani et al., 2021) that reported elevated internalizing or externalizing problems during initial lockdowns, compared to pre-pandemic, in the UK, Italy, and the Netherlands. The present findings extend prior work by showing that children continue to experience elevated mental health difficulties while not in lockdown (only 26.5% of the sample was in quarantine at the time of the survey) and several months after the pandemic first began. In contrast, prior work did not find significant increases in mental health during the pandemic in a sample of Hispanic adolescents between April-May 2020 in the USA (Penner et al., 2020). Several characteristics of the sample (age, ethnicity) and study methodology (e.g., timing) could explain these distinct findings. As the COVID-19 pandemic extends into a second year, the disruptions to children's daily lives are expected to continue, including additional school closures, reduced mental health support services, cancellation of extra-curricular activities, and isolation from friends and extended family. Children who initially adapted well to the pandemic might begin to experience adverse outcomes as these restrictions persist. Thus, there is a crucial need for continued close monitoring of children's adjustment as the ramifications of the pandemic continue to unfold.

This study further identifies distinct correlates of child internalizing and externalizing problems. Maternal hostility during the pandemic was uniquely associated with elevated child externalizing behavior, whereas maternal anxiety was uniquely associated with elevated internalizing behaviors, after accounting for COVID-specific stress and other risk factors. These findings are in line with extensive literature showing that parental mental health and non-optimal parenting behavior are risk factors for child mental health problems (Goodman et al., 2011; Achtergarde et al., 2015; Ryan et al., 2017). Importantly, parents are experiencing high levels of stress and mental health challenges during the pandemic (Cameron et al., 2020; Adams et al., 2021), which can impact the quality of parent-child relationships (Chung et al., 2020) and have a direct effect on child adjustment.

The unique effect of parental hostility on externalizing problems and anxiety on internalizing problems is also supported by prior research. A prior study found that parental depression and anxiety were specially associated with child internalizing problems and parent hostility was associated with more child externalizing problems during the pandemic (Whittle et al., 2020). Additional research, not in the context of the COVID-19 pandemic, strongly connects maternal internalizing symptoms with child internalizing symptoms (e.g., Goodman et al., 2011) and negative/hostile parenting behavior with child externalizing problems (Gershoff, 2002; Murray et al., 2007, 2008). Social modeling of specific anxious or hostile behaviors may contribute to these unique associations. It is also possible that in the current small sample, these were the only associations with the strength to reach significance. Future research is needed to examine the unique effect of parenting and parent mental health on changes in child mental health.

This research highlights an urgent need for mental health support for children and parents, particularly as the COVID-19 pandemic progresses and the burden on families intensifies. Ensuring the availability of essential resources to families is a crucial first step in limiting the impact on child mental health. For example, ongoing access to financial support and assistance with homeschooling will be vital in reducing parenting stress, strengthening parent-child relationships, and improving both parent and child mental health. In addition, bolstering other supports for parents, including easily accessible mental health and stress reduction programs, will limit the impact of pandemic-related stress on parent mental health and the associated impact on children.

### Future Directions

Continued longitudinal work is essential to determine the long-term effects of this pandemic on child mental health. Longitudinal studies that include multiple time points will permit the assessment of mechanisms (e.g., social isolation, parenting behavior) that account for elevations in child mental health. Intervention efforts can then focus on targeting these mechanisms to improve child mental health. In addition, studies with larger samples are necessary to examine child and parent characteristics that act as risk/protective factors. The ongoing restrictions aimed to slow the spread of COVID-19 are likely to intensify economic and social disparities, resulting in adverse outcomes for the most vulnerable children. Future work is needed on diverse samples, particularly with children who have pre-existing risk factors (e.g., learning difficulties, history of mental health problems, lower SES), to understand how to best serve children who face an elevated risk of mental health problems.

### Limitations

The current findings must be considered in light of study limitations. First, this study had a relatively small sample size. It is possible that additional significant associations (between parenting and child internalizing problems, or parent mental health and child externalizing problems) would be detected in a larger sample. Future work with larger samples is needed to replicate and generalize these findings. Second, this is a low-risk (moderate to high SES) sample. Related, children in this sample were relatively low risk in terms of the clinical severity of their symptoms. Nonetheless, mental health symptoms increased significantly and meaningfully, suggesting that even children at relatively low risk have been negatively impacted by the pandemic. Third, this study relied exclusively on maternal report. In addition, we were not able to assess changes in parent mental health and changes in parenting behavior in this work, given that comparable measures were not captured before the pandemic. Future multi-method (questionnaire and observation) and multi-informant assessments are needed to gain a more diverse understanding of the correlates of changes in parent and child mental health during the pandemic.

### Summary

This study augments prior research by providing longitudinal evidence that children (7 to 9 years old) are experiencing *elevated* internalizing and externalizing problems during the COVID-19 pandemic compared to before the pandemic. Further, maternal anxiety was associated with a greater increase in child internalizing symptoms, and maternal hostility was associated with a greater increase in child externalizing symptoms. These findings highlight the need for widely available mental health supports and interventions for families to limit the mental health burden on parents and children during the COVID-19 pandemic.

## Data Availability Statement

The raw data supporting the conclusions of this article will be made available by the authors, without undue reservation.

## Ethics Statement

The studies involving human participants were reviewed and approved by Hamilton Integrated Research Ethics Board (Project #10816). The patients/participants provided their written informed consent to participate in this study.

## Author Contributions

JK conceptualized and designed the study, oversaw data collection, conducted data analyses, drafted the initial manuscript, and reviewed and revised the manuscript. AG conceptualized and designed the study, reviewed and revised the manuscript. HK assisted with data collection, contributed to writing sections of the manuscript, and reviewed and revised the manuscript. All authors contributed to the article and approved the submitted version.

## Conflict of Interest

The authors declare that the research was conducted in the absence of any commercial or financial relationships that could be construed as a potential conflict of interest.

## Publisher's Note

All claims expressed in this article are solely those of the authors and do not necessarily represent those of their affiliated organizations, or those of the publisher, the editors and the reviewers. Any product that may be evaluated in this article, or claim that may be made by its manufacturer, is not guaranteed or endorsed by the publisher.

## Footnotes

^1^Of the 68 participants, 67 completed the follow-up survey between May 28 and August 10, 2020; one participant completed the follow-up survey in November 2020.

^2^When comparing Z scores, based on the grand mean and standard deviation of the BPM and CBCL scores, there were significant differences in internalizing problems (*t*_(63)_ = −6.81, *p* < 0.001) and externalizing problems (*t*_(63)_ = −9.67, *p* < 0.001) before and during the pandemic.

## References

[B1] Achenbach T. M. McConaughy S. H. Ivanova M. Y. Rescorla L. A. (2011). Manual for the ASEBA Brief Problem Monitor (BPM). University of Vermont, Research Center for Children, Youth, and Families, Burlington, United States.

[B2] Achenbach T. M. Ruffle T. M. (2000). The child behavior checklist and related forms for assessing behavioral/emotional problems and competencies. Pediatr. Rev. 21, 265–271. 10.1542/pir.21-8-265 10922023

[B3] Achterberg M. Dobbelaar S. Boer O. D. Crone E. A. (2021). Perceived stress as mediator for longitudinal effects of the COVID-19 lockdown on wellbeing of parents and children. Sci. Rep. 11:2971. 10.1038/s41598-021-81720-8 33536464 PMC7859207

[B4] Achtergarde S. Postert C. Wessing I. Romer G. Müller J. M. (2015). Parenting and child mental health: influences of parent personality, child temperament, and their interaction. Fam. J. 23, 167–179. 10.1177/1066480714564316

[B5] Adams E. Smith D. Caccavale L. J. Bean M. K. (2021). Parents are stressed! Patterns of parent stress across COVID-19. Front. Psychiatry, 12, 300–310. 10.3389/fpsyt.2021.626456 33897489 PMC8060456

[B6] Andresen E. M. Malmgren J. A. Carter W. B. Patrick D. L. (1994). Screening for depression in well older adults: evaluation of a short form of the CES-D (Center for Epidemiologic Studies Depression Scale). Am. J. Prev. Med. 10, 77–84. 10.1016/S0749-3797(18)30622-6 8037935

[B7] Ara E. (2016). Internalizing and externalizing problems in adolescents analyzing the gender difference. Int. J. Sci. Res. 6, 328–337.

[B8] Bignardi G. Dalmaijer E. S. Anwyl-Irvine A. L. Smith T. A. Siugzdaite R. Uh S.. (2020). Longitudinal increases in childhood depression symptoms during the COVID-19 lockdown. Arch. Dis. Child. archdischild-2020-320372. 10.31219/osf.io/v7f3q. [Epub ahead of print]. 33298552 PMC7733224

[B9] Bikmazer A. Kadak M. T. Görmez V. Dogan U. Aslankaya Z. D. Bakir F.. (2020). Parental psychological distress associated with COVID-19 outbreak: a large-scale multicenter survey from Turkey. Int. J. Soc. Psychiatry. 10.1177/0020764020970240. [Epub ahead of print]. 33148091

[B10] Calvano C. Engelke L. Di Bella J. Kindermann J. Renneberg B. Winter S. M. (2021). Families in the COVID-19 pandemic: parental stress, parent mental health and the occurrence of adverse childhood experiences-results of a representative survey in Germany. Eur. Child. Adolesc. Psychiatry 1–13. 10.1007/s00787-021-01739-0. [Epub ahead of print]. 33646416 PMC7917379

[B11] Cameron E. E. Joyce K. M. Delaquis C. P. Reynolds K. Protudjer J. L. P. Roos L. E. (2020). Maternal psychological distress & mental health service use during the COVID-19 pandemic. J. Affect. Disord. 276, 765–774. 10.1016/j.jad.2020.07.081 32736186 PMC7370903

[B12] Cantiani C. Dondena C. Capelli E. Riboldi E. M. Molteni M. Riva V. (2021). Effects of COVID-19 lockdown on the emotional and behavioral profiles of preschool Italian children with and without familial risk for neurodevelopmental disorders. Brain Sci. 11, 477–491. 10.3390/brainsci11040477 33918593 PMC8070543

[B13] Chung G. Lanier P. Wong P. Y. J. (2020). Mediating effects of parental stress on harsh parenting and parent-child relationship during coronavirus (COVID-19) pandemic in Singapore. J J. Fam. Viol. 1–12. 10.1007/s10896-020-00200-1 32895601 PMC7467635

[B14] Cohen S. Williamson G. (1988). Perceived stress in a probability sample of the United States, in The Social Psychology of Health, eds S. Spacapan and S. Oskamp (Newbury Park, CA: Sage), 31–68.

[B15] Connell C. M. Strambler M. J. (2021). Experiences with COVID-19 stressors and parents' use of neglectful, harsh, and positive parenting practices in the Northeastern United States. Child. Maltreat. 10775595211006465. 10.1177/10775595211006465. [Epub ahead of print]. 33787377 PMC9218961

[B16] Crescentini C. Feruglio S. Matiz A. Paschetto A. Vidal E. Cogo P.. (2020). Stuck outside and inside: an exploratory study on the effects of the COVID-19 outbreak on Italian parents and children's internalizing symptoms. Front. Psychol. 11:586074. 10.3389/fpsyg.2020.586074 33192917 PMC7649806

[B17] Cusinato M. Iannattone S. Spoto A. Poli M. Moretti C. Gatta M.. (2020). Stress, resilience, and well-being in Italian children and their parents during the COVID-19 pandemic. Int. J. Environ. Res. Public. Health 17:8297. 10.3390/ijerph17228297 33182661 PMC7696524

[B18] Daly M. Sutin A. R. Robinson E. (2021). Depression reported by US adults in 2017–2018 and March and April 2020. J. Affect. Disord. 278, 131–135. 10.1016/j.jad.2020.09.065 32956962 PMC7490280

[B19] Davidson B. Schmidt E. Mallar C. Mahmoud F. Rothenberg W. Hernandez J.. (2020). Risk and resilience of well-being in caregivers of young children in response to the COVID-19 pandemic. Transl. Behav. Med. 11, 305–313. 10.1093/tbm/ibaa124 33236766 PMC7890655

[B20] Fox J. (2015). Applied Regression Analysis and Generalized Linear Models. Los Angeles: SAGE.

[B21] Francisco R. Pedro M. Delvecchio E. Espada J. P. Morales A. Mazzeschi C.. (2020). Psychological symptoms and behavioral changes in children and adolescents during the early phase of COVID-19 quarantine in three European Countries. Front. Psychiatry. 11:570164. 10.3389/fpsyt.2020.570164 33343415 PMC7744455

[B22] Gershoff E. T. (2002). Corporal punishment by parents and associated child behaviors and experiences: a meta-analytic and theoretical review. Psychol. Bull. 128, 539–579. 10.1037/0033-2909.128.4.539 12081081

[B23] Goodman S. H. Rouse M. H. Connell A. M. Broth M. R. Hall C. M. Heyward D. (2011). Maternal depression and child psychopathology: a meta-analytic review. Clin. Child. Fam. Psychol. Rev. 14, 1–27. 10.1007/s10567-010-0080-1 21052833

[B24] Janssen L. H. C. Kullberg M. J. Verkuil B. van Zwieten N. Wever M. C. M. van Houtum L. A. E. M.. (2020). Does the COVID-19 pandemic impact parents' and adolescents' well-being? an EMA-study on daily affect and parenting. PLoS. ONE. 15:e0240962. 10.1371/journal.pone.0240962 33064778 PMC7567366

[B25] Johnston D. W. Schurer S. Shields M. A. (2013). Exploring the intergenerational persistence of mental health: evidence from three generations. J. Health. Econ. 32, 1077–1089. 10.1016/j.jhealeco.2013.09.001 24103501

[B26] Khoury J. E. Atkinson L. Bennett T. Jack S. M. Gonzalez A. (2021). COVID-19 and mental health during pregnancy: the importance of cognitive appraisal and social support. J. Affect. Disord. 282, 1161–1169. 10.1016/j.jad.2021.01.027 33601691 PMC7837227

[B27] Lee S. J. Ward K. P. Lee J. Y. Rodriguez C. M. (2021). Parental social isolation and child maltreatment risk during the COVID-19 pandemic. J. Fam. Violence 1–12. 10.1007/s10896-020-00244-3. [Epub ahead of print]. 33462526 PMC7807402

[B28] Lovejoy M. C. Weis R. O'Hare E. Rubin E. C. (1999). Development and initial validation of the parent behavior inventory. Psychol. Assess. 11, 534–545. 10.1037/1040-3590.11.4.534

[B29] Marchetti D. Fontanesi L. Di Giandomenico S. Mazza C. Roma P. Verrocchio M. C. (2020). The effect of parent psychological distress on child hyperactivity/inattention during the COVID-19 lockdown: testing the mediation of parent verbal hostility and child emotional symptoms. Front. Psychol. 11:567052. 10.3389/fpsyg.2020.567052 33362632 PMC7758226

[B30] McLeod B. D. Wood J. J. Weisz J. R. (2007). Examining the association between parenting and childhood anxiety: a meta-analysis. Clin. Psychol. Rev. 27:155–172. 10.1016/j.cpr.2006.09.002 17112647

[B31] Murray L. Cooper P. J. Creswell C. Schofield E. Sack C. (2007). The effects of maternal social phobia on mother- infant interactions and infant social responsiveness. J. Child. Psychol. Psychiatry 48, 45–52. 10.1111/j.1469-7610.2006.01657.x 17244269

[B32] Murray L. De Rosnay M. Pearson J. Bergeron C. Schofield L. Royal-Lawson M.. (2008). Intergenerational transmission of maternal social anxiety: the role of the social referencing process. Child. Dev. 79, 1049–1064. 10.1111/j.1467-8624.2008.01175.x 18717906

[B33] Negriff S. Susman E. J. (2011). Pubertal timing, depression, and externalizing problems: a framework, review, and examination of gender differences. J. Res. Adolesc. 21, 717–746. 10.1111/j.1532-7795.2010.00708.x

[B34] Orgilés M. Morales A. Delvecchio E. Mazzeschi C. Espada J. P. (2020). Immediate psychological effects of the COVID-19 quarantine in youth from Italy and Spain. Front. Psychol. 11:579038. 10.3389/fpsyg.2020.579038 33240167 PMC7677301

[B35] Patrick S. W. P. Henkhaus L. E. Zickafoose J. S. Lovell K. Halvorson A. Loch S.. (2020). Well-being of parents and children during the COVID-19 pandemic: a national survey. Pediatrics 4:146. 10.1542/peds.2020-016824 32709738

[B36] Penelo E. De la Osa N. Navarro J. B. Domènech J. M. Ezpeleta L. (2017). The Brief Problem Monitor-Parent form (BPM-P), a short version of the child behavior checklist: psychometric properties in Spanish 6-to 8-year-old children. Psychol. Assess. 29, 1309–1320. 10.1037/pas0000428 28080108

[B37] Penner F. Ortiz J. H. Sharp C. (2020). Change in youth mental health during the COVID-19 pandemic in a majority Hispanic/Latinx US sample. J. Am. Acad. Child. Adolesc. Psychiatry 60, 513–523. 10.1016/j.jaac.2020.12.027 33359408

[B38] Pierce M. Hope H. Ford T. Hatch S. Hotopf M. John A.. (2020). Mental health before and during the COVID-19 pandemic: a longitudinal probability sample survey of the UK population. Lancet. Psychiatry 7, 883–892. 10.1016/S2215-0366(20)30308-4 32707037 PMC7373389

[B39] Piper B. J. Gray H. M. Raber J. Birkett M. A. (2014). Reliability and validity of brief problem monitor, an abbreviated form of the child behavior checklist. Psychiatry Clin. Neurosci. 68, 759–767. 10.1111/pcn.12188 24735087 PMC4182328

[B40] Prime H. Wade M. Gonzalez A. (2020). The link between maternal and child verbal abilities: an indirect effect through maternal responsiveness. Dev. Sci. 23:e12907. 10.1111/desc.12907 31571333

[B41] Racine N. Korczak D. J. Madigan S. (2020). Evidence suggests children are being left behind in COVID-19 mental health research. Eur. Child. Adolesc. Psychiatry. 5, 1–2. 10.1007/s00787-020-01672-8 33151431 PMC7643721

[B42] Rueger S. Y. Katz R. L. Risser H. J. Lovejoy M. C. (2011). Relations between parental affect and parenting behaviors: a meta-analytic review. Parent. Sci. Pract. 11, 1–33. 10.1080/15295192.2011.539503

[B43] Ryan R. O'Farrelly C. Ramchandani P. (2017). Parenting and child mental health. London. J. Prim. Care. 9, 86–94. 10.1080/17571472.2017.1361630 PMC5694794 29181091

[B44] Spitzer R. L. Kroenke K. Williams J. B. Löwe B. (2006). A brief measure for assessing generalized anxiety disorder: the GAD-7. Archiv. Intern. Med. 166, 1092–1097. 10.1001/archinte.166.10.1092 16717171

[B45] Stone L. L. Mares S. H. Otten R. Engels R. C. Janssens J. M. (2016). The co-development of parenting stress and childhood internalizing and externalizing problems. J. Psychopathol. Behav. Assess. 38, 76–86. 10.1007/s10862-015-9500-3 27069304 PMC4789299

[B46] Tompkins T. L. Hockett A. R. Abraibesh N. Witt J. L. (2011). A closer look at co-rumination: gender, coping, peer functioning and internalizing/externalizing problems. J. Adolesc. 34, 801–811. 10.1016/j.adolescence.2011.02.005 21411134

[B47] United Nations (2020). Policy Brief: the Impact of COVID-19 on Children. New York, NY: UN Headquarters.

[B48] Wade M. Prime H. Browne D. T. (2020). Why we need longitudinal mental health research with children and youth during (and after) the COVID-19 pandemic. Psychiatry Res. 290:113143. 10.1016/j.psychres.2020.113143 32502829 PMC7253952

[B49] Whittle S. Bray K. O. Lin S. Schwartz O. (2020). Parenting and child and adolescent mental health during the COVID-19 pandemic. PsyArXiv. 10.31234/osf.io/ag2r7. [Epub ahead of print].33110413

[B50] Xie X. Xue Q. Zhou Y. Zhu K. Liu Q. Zhang J.. (2020). Mental health status among children in home confinement during the coronavirus disease 2019 outbreak in Hubei Province, China. JAMA. Pediatr. 174, 898–900. 10.1001/jamapediatrics.2020.1619 32329784 PMC7182958

[B51] Yap M. B. Pilkington P. D. Ryan S. M. Jorm A. F. (2014). Parental factors associated with depression and anxiety in young people: a systematic review and meta-analysis. J. Affect. Disord. 156, 8–23. 10.1016/j.jad.2013.11.007 24308895

